# Pathomorphological and microbiological studies in sheep with special emphasis on gastrointestinal tract disorders

**DOI:** 10.14202/vetworld.2015.1015-1020

**Published:** 2015-08-26

**Authors:** Sarvan Kumar, K. K. Jakhar, Vikas Nehra, Madan Pal

**Affiliations:** 1 Department of Veterinary Pathology, Lala Lajpat Rai University of Veterinary & Animal Sciences, Hisar, Haryana, India; 2 Department of Veterinary Surgery and Radiology, Lala Lajpat Rai University of Veterinary & Animal Sciences, Hisar, Haryana, India

**Keywords:** gastrointestinal tract disorders, *in-vitro* chemotherapeutic sensitivity, microbiology, pathomorphology, sheep

## Abstract

**Aim:**

The present study was envisaged to elucidate the pathomorphological and microbiological aspects of gastrointestinal tract (GIT) disorders of sheep/lambs.

**Materials and Methods:**

Samples for research were collected from 12 sheep died with a history of GIT disorders which were brought for post-mortem examination to the Department of Veterinary Pathology, Lala Lajpat Rai University of Veterinary and Animal Sciences, Hisar, for pathomorphological and microbiological examination.

**Results:**

Gross pathological changes in various organs noticed were abomasitis, congestion and hemorrhages in intestine; necrotic foci on liver surface; enlarged, hard, and indurated mesenteric lymph nodes, hydropericardium, congestion, hemorrhages and consolidation of lungs and congestion and soft kidneys as the major change. On histopathological examination, there were abomasitis with leukocyte infiltration, enteritis with desquamation of mucosal epithelium and goblet cell hyperplasia, lymphadenitis with depletion of lymphocytes in the germinal center of lymphoid follicle, and splenitis with depletion of lymphocytes in the white pulp. In the liver congestion, degenerative changes in hepatocytes including cloudy swelling, fatty changes, congestion in sinusoids, and dilatation of sinusoids leading to atrophy of hepatocytes. Lungs evidenced edema, congestion, emphysema, serous inflammation, thickening of interlobular septa, fibrinous pleuritis, and peribronchiolar lymphoid follicle formation. Heart revealed sarcocystosis, fibrinous pericarditis, and hyalinization of the myocardium. In kidneys, congestion, focal interstitial nephritis, hyaline degeneration, and coagulative necrosis were seen. For microbiological aspects; cultural isolation was done from samples of liver, abomasum, mesenteric lymph nodes, spleen, heart blood, lungs, and kidneys from the carcasses of sheep/lambs. *Escherichia coli* was the only bacterium isolated during present studies. *E. coli* isolates from different tissues of carcasses of sheep/lambs were subjected to *in-vitro* drug sensitivity testing. Ciprofloxacin, cefixime, polymyxin B, amoxicillin + sulbactam, and amoxicillin + clavulanic acid were the most sensitive drugs followed by amikacin, ofloxacin, ampicillin, co-trimoxazole, and amoxicillin.

**Conclusions:**

From the present study, it is reasonable to conclude that the major etiopathological cause of GIT disorders in sheep was *E. coli* infection, which causes a pathomorphological effect on various cadaver organs *viz*. abomasum, intestine, liver, mesenteric lymph nodes, lungs, spleen, kidneys, and heart followed by parasitic infection of *Haemonchus contortus*.

## Introduction

Agriculture, with its allied sectors such as animal husbandry, is unquestionably the largest livelihood provider in India, more so in the vast rural areas. It also contributes about 15.18% to the gross domestic product [1]. According to the estimates of the Central Statistical Organization, the value of output from livestock and fisheries sectors together at current prices was about 31.6% of the value of output from agriculture and allied sectors. Diseases among these animals cause heavy economic loss. India ranks second in sheep population (71.56 million) in the world [2]. Of this, 0.63 million sheep account from Haryana [3].

Gastrointestinal tract (GIT) disorders are arguably the most important causes of suboptimal productivity in sheep rearing, albeit that often occurs concurrently with other problems. The principal reason for keeping farmed sheep is to convert primary forage, herbage or cereal crops into a marketable product. The efficiency of conversion of feed to meat is greater in sheep that achieve maximum growth rates than in ill-thrifty animals, because there is a daily feed requirement that must be met before the growth can occur, irrespective of the time taken to reach slaughter weight.

A number of viral, bacterial, and parasitic disease result in morbidity and mortality and decline in overall production. The diseases most commonly associated with GIT lesions are Johne’s disease, *Escherichia coli* infection, Salmonellosis, enterotoxemia caused by *Clostridium perfringens* Type-B, C, and D, peste des petits ruminants, parasitism, tympany, and others diarrheic disorders. Parasitism particularly by Helminths, impairs health by causing inappetence, diarrhea, ­anemia, and in severe case death. Therefore, the profitability of global sheep farming is heavily influenced by the efficiency of feed conversion to meat; control of GIT disorder is a prerequisite for the economically sustainable product. To define the cause of death, complete post-mortem is a must. For this purpose, current studies were planned on the pathomorphological and microbiological investigation of GIT disorders in sheep.

## Materials and Methods

### Ethical approval

Not necessary as study based on post-mortem examination only. Samples for research were collected from the 12 sheep/lambs brought for post-mortem examination to the Department of Veterinary Pathology, Lala Lajpat Rai University of Veterinary and Animal Sciences, Hisar, for pathomorphological and microbiological examination irrespective of age, sex, and breed.

### Pathomorphological examination

Gross changes: All the tissues/organs of the carcasses were examined critically for the presence of gross pathological alterations and the lesions so detected were recorded.

### Histopathology

Small piece of organs showing lesions such as abomasum, intestine, mesenteric lymph nodes, liver, spleen, heart, lungs, and kidneys were collected in 10% buffered formalin for histopathological studies. The fixed tissues were washed in running tap water overnight, dehydrated in acetone, cleared in benzene, and embedded in paraffin wax (melting point 60-62°C). Paraffin sections were cut at the thickness of 4-5 µ and staining is done using Lily Mayer’s hematoxylin and 2% water soluble eosin [4].

### Microbiological examination

During post-mortem examination, tissue and heart blood samples for bacteriological studies were collected aseptically in sterile Petri dishes and sterile disposable syringe, respectively. Small pieces up to 0.5-1 cm thickness of internal organs showing lesions *viz*. liver, mesenteric lymph nodes, lungs, spleen, kidneys, and heart blood (near about 2 ml) were collected without any anticoagulant from each case for bacteriological examination. Then samples from these organs and heart blood were inoculated on nutrient agar (NA) and Mc-Conkey’s lactose agar (MLA) and eosin methylene blue agar (EMB) selective media plates by streak plate technique on same day of sample collection. The plates were then incubated aerobically at 37°C for 24-48 h and examined for the presence of growth, if any. Colony characteristics were noted from NA and MLA plate. All the different type of colonies developed were obtained in pure culture. The pure cultures so obtained were subjected to a biochemical test for further characterization. Identification of all isolates was done following the procedures of Quinn *et al*. [5]. All the organisms so obtained were stained by Gram’s staining and examined for their morphological characters.

### *In-vitro* chemotherapeutic sensitivity

Isolates were subjected to *in-vitro* drug sensitivity testing using 10 antimicrobials by the single disc diffusion method following the method of Bauer *et al*. [6]. Ciprofloxacin (30 mcg), cefixime (5 mcg), polymyxin B (300 units/disc), amoxicillin + sulbactam (30/15 mcg), amoxicillin + clavulanic acid (20/10 mcg), amikacin (30 mcg), ofloxacin (5 mcg), ampicillin (25 mcg), co-trimoxazole (1.25 mcg), and amoxicillin (30 mcg) were used for drug sensitivity pattern. Results were recorded for the present sensitivity of microbial agent.

### Parasitological examination

During post-mortem examination; rule out every possibility of internal parasitic infection along with fecal contents of abomasum and small intestine. The severity of infection was ascertained by egg count and 150 eggs/g and more were considered as a severe infection.

## Results

**Figure-1:**
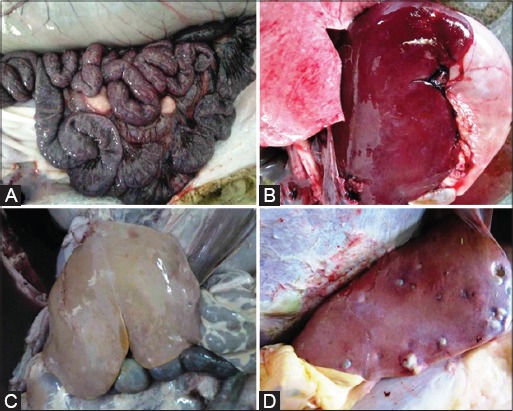
Haemorrhagic enteritis and enlargement of mesenteric lymph node, B: Severe congestion in liver, C: Fibrinous perihepatitis, D: Small abscess on liver.

The gross pathological changes observed in different organs were congestion, petechial to ecchymotic hemorrhage, necrotic foci, hypertrophy, catarrhal and hemorrhagic inflammation, consolidation and tubercle formation, abscess and cyst in different vital organs. In abomasum, congestion was the most prominent change (n=5). In the small intestine, major changes observed were congestion (8 cases) followed by catarrhal and hemorrhagic enteritis (n=4) as shown in Figure-1a. Major gross changes in the lymph node were enlargement, firmness, and induration (n=2). In liver, most prominent change was congestion (n=4) followed by necrotic foci (n=3), fibrinous perihepatitis (Figure-1b and c) along with enlarged gall bladder due to thick bile and abscess in liver (Figure-1d), respectively. Spleen revealed congestion (n=2) and petechial hemorrhages in parenchyma as the major change observed in case of spleen. In the heart, major changes observed were hydropericardium (n=2) followed by congestion (n=3) and hemorrhages in the endocardium. In lungs, the most prominent change was congestion (n=7) followed by emphysema (n=4) and consolidation (n=3). In two cases, formation of small tubercle was also seen in the diaphragmatic lobe. In kidneys, congestion (n=3), soft kidneys (n=3), mottled kidneys, and cyst on kidneys were the major changes observed. When fecal contents of abomasum and small intestine were observed for ruling out parasitic infection; it reveled large number of thread like organism in five cases (n=5) which were confirmed as *Haemonchus contortus*.

**Figure-2:**
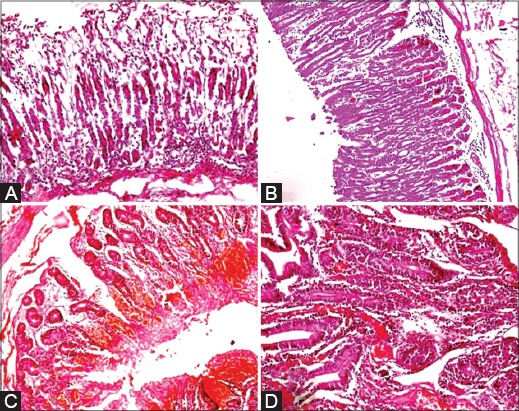
A: Abomasum: Leucocytic infiltration in mucosa (H. & E. × 100), B: Abomasum: Initiation of lymphoid follicle in submucosa (H. & E. × 100), C: Intestine: Severe haemorrhagic enteritis (H. & E. × 100), D: Intestine: Tendency of replacing the glandular epithelium with infiltrating cells, (H. & E. × 200).

**Figure-3:**
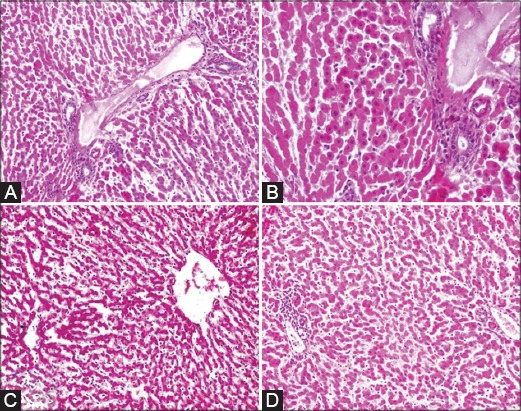
A: Liver: Focal area of coagulative necrosis and increased no. of bile duct in portal triad area (H. & E. × 200), B: Liver: Various stages of necrosis in hepatocytes in periportal area. (H. & E. × 400), C: Liver: Telangiectasis along with atrophy of hepatic cords. (H. & E. × 100), D: Liver: Coagulative necrosis in parenchyma and lymphoid follicle formation in portal triad area (H. & E. × 200).

**Figure-4:**
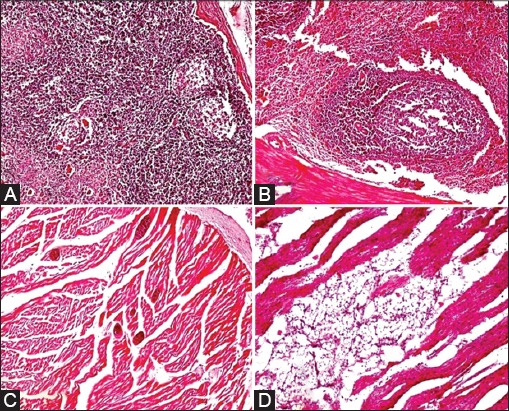
A: Mesentric lymph node: Depletion of lymphocytes in germinal centre in cortex of lymph node (H. & E. × 100), B: Spleen: Depletion of lymphocyte in white pulp area (H. & E. × 100), C: Heart: Pericardial fibrinous layer and severe sarcocystosis in myocardium (H. & E. × 100), D: Heart: Fat necrosis in cardiac muscle (H. & E. × 200).

The histopathological lesions of various organs are depicted in Figures-2-4. In abomasum, edema, congestion, and leukocytic infiltration in mucosa and submucosa with a tendency to lymphoid follicle formation were observed (Figure-2a and b). In the small intestine, major microscopic lesions were edema, mild congestion in mucosa and submucosa, and hemorrhages in the mucosa (Figure-2c and d). Other changes were goblet cell hyperplasia, necrosis and replacement of glands of Leiberkuhn’s by mononuclear cells, and desquamation of mucosal epithelium. There was also depletion of lymphocytes in Payer’s patches in the large intestine. In the liver, major microscopic lesions were congestion in portal triad area, leukocytic infiltration, and mild bile duct hyperplasia. Congestion of central vein with cloudy swelling and fatty changes in hepatocytes were also observed. Bacterial rods were also seen below the capsule, sinusoids with varying degree of degenerative changes in hepatocytes. Focal area of coagulative necrosis in parenchyma in the periportal area (Figure-3a) and various stages of necrosis such as pyknosis, karyorrhexis, and karyolysis were also noticed (Figure-3b). Other changes were telangiectasias along with atrophy of hepatic cords (Figure-3c). Necrosis in parenchyma and bile duct hyperplasia along with fibroblast proliferation were found. Initiation of lymphoid follicle formation along with degenerative and necrotic changes in parenchyma was seen (Figure-3d). Mesenteric lymph nodes when examined microscopically, revealed edema, congestion, and mild leukocytic infiltration in the capsule. There was depletion of lymphocytes in germinal centers of cortex (Figure-4a) and excess of mononuclear cells in medullary sinuses of the medulla. Spleen revealed mild to severe depletion of lymphocytes in white pulp area (Figure-4b) and reticulo-endothelial cell hyperplasia around the white pulp area. In the heart, edema and congestion were seen in the myocardium along with leukocytic infiltration in some cases. Severe hemorrhages in the epicardium and serous inflammation in the myocardium were evidenced. There was one case of fibrinous pericarditis and sarcocystosis in the myocardium (Figure 4c). At places, fat necrosis was seen in myocardium (Figure-4d). Hyalinization of myocardium, edema, and focal area of mild necrosis along with mononuclear cells infiltration were also observed. In lungs, histopathological lesions revealed edema, congestion, emphysematous alveoli, serous inflammation, thickened interlobular septa due to serofibrinous exudates and infiltration of mononuclear cells in the peribronchiolar area and hyperplasia bronchiolar epithelium. In the heart, there was pleuritis along with thickening of pleura and peribronchiolar lymphoid follicle formation along with serous inflammation. In kidneys, there was congestion of intertubular blood vessels and coagulative necrosis along with leukocytic infiltration. Focal areas of necrosis along with hyaline materials in tubules and interstitial nephritis with infiltration of the mononuclear cell were observed.

**Figure-5:**
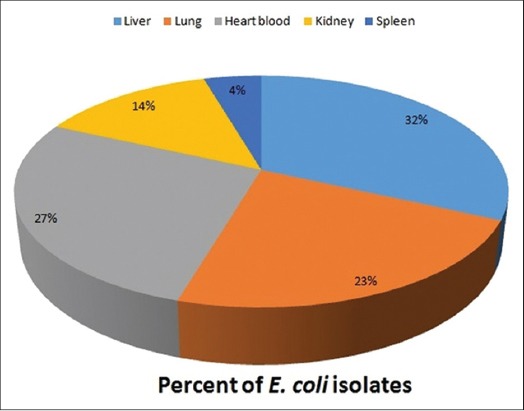
Organwise percent of *E. coli* isolates.

Out of 72 representative tissues which were collected for microbiological examination; 22 samples produced pink colonies on MLA and creamy white mucoid/non-mucoid colonies on NA. Gram-staining revealed pink colored rods and Catalase test further confirmed Gram-negative bacteria. Metallic sheen was noticed in all these cases on EMB agar. After performing biochemical tests (oxidation-fermentation test, nitrate test, H2S production on triple sugar iron medium, indole, methyl red, Voges–Proskauer, citrate utilization test, and urease test) and culture characteristics revealed isolates positive for *E. coli*. The results of *E. coli* bacteria isolated from different organs of carcasses of sheep/lambs are depicted in Figure-5. Out of 22 isolates of *E. coli*., organwise isolates were 7 from liver (31.81%), 5 from lung (22.72%), 6 from heart blood (27.27%), 3 from kidney (13.64%), and 1 from spleen (4.5%). No bacteria were isolated from mesenteric lymph nodes. All the isolates of *E. coli* showed maximum sensitivity against cefixime, ciprofloxacin, polymyxin-B, amoxicillin + sulbactam, and amoxicillin + clavulanic acid (each 100%) followed by amikacin and ofloxacin (90%). Least sensitivity was observed to ampicillin, co-trimoxazole, and amoxicillin (70%).

## Discussion

Abomasitis, catarrhal, and hemorrhagic enteritis may be due to gastrointestinal parasitism (*H. contortus*) and various bacterial and viral infections. Hemorrhagic enteritis may be because of simultaneous infection of rotavirus and *E. coli*. [7]. Rotavirus is the leading etiological agent of acute viral gastroenteritis in young ones of many species of animals. Rotavirus damages microvilli of the small intestine, which causes reduced starch digestion, results in promotion of excess bacterial (*E. coli*) growth simultaneously. It induces an osmotic effect resulting in diarrhea. In lambs, rotavirus enteritis is more severe when complicated by simultaneous infection with enterotoxigenic *E. coli*. [8]. A wide range of infectious and parasitic agents that produces myocarditis and include suppurative, necrotic, hemorrhagic, lymphocytic, and eosinophilic response. Eosinophilic myocarditis in current studies may result of sarcocystosis. Hydropericardium observed in lambs can be correlated with heavy infection of blood sucking stomach worms resulting in excessive loss of serum albumin decreasing the osmotic pressure of plasma which forces fluid to move out of blood to tissues and accumulation of fluid in pericardial sac resulting in hydropericardium [9,10]. In the present study, infection of *H. contortus* may be due to the development of resistant strain [11]. Soft kidneys may be correlated with overeating of pasture results in the accumulation of undigested starch which may enhance proliferation of *Clostridium perferingens* Type D bacteria in rapidly growing lambs. These bacteria grow in feed in upper intestine and secrete two toxins that are absorbed by the gut, alpha toxin damages gut lining, and epsilon toxin absorbed by body and causes damage to brain, heart, kidneys, and other tissues. Chronic passive congestion leads to persistent hypoxia in the centrilobular area and because of oxygen and nutrient, deprivation leads to degeneration and hepatocyte atrophy. As a result, sinusoids in these areas are dilated which leads to telangiectasias [12]. Mesenteric lymph nodes when examined microscopically, revealed edema, congestion, and mild leukocytic infiltration in capsule [13].

Microbiological finding were in consonance of studies conducted by Kumar *et al*. [14]. They concluded that out of 72 tissue samples 48 were confirmed for *E. coli*. Out of 48 isolates, only 18 were serotyped and out of these 13 belonged to ‘O’ serogroup whereas remaining five isolate were untypable. The most prevalent serotype were O168 (5) followed by O 60 (4), O1 (1), O91 (1), 0102 (1), and O116 (1). A relatively high rate of *E. coli* infection in sheep/lambs in the present study might be due to errors in colostrum feeding, inefficient production of antibodies, stress, and hormonal changes which enhance the growth of opportunistic bacteria and therefore, infection flare up. Sharma and Pruthi [15] reported that *E. coli* as the most predominant bacterial organism in GIT disorders of sheep carcasses followed by *Proteus* spp., *Klebsiella* spp., *Salmonella typhimurium* 4,5:i:1,2, *Staphylococcus* spp. and *Pseudomonas aeruginosa*. The *E. coli* strains belonged to 14 different serotypes, of these, 52.3% were of 7 serotypes O172 (7), O158 (14), O88 (17), O44 (7), O153 (17), O22 (11), and O25 (10). Other serotypes were O32 (1), O132 (2), O64 (1), O42 (5), O156 (5), O70 (1), and O91 (3). Three strains were rough and 21 remained untyped. More or less similar findings were revealed by Tibbo *et al*. [16] and Lashari and Tasawar [17], Sharma and Pruthi [15].

In the present investigation, anti-biogram patterns of different bacterial organisms isolated from carcasses of sheep/lambs showed varying degree of sensitivity to the chemotherapeutic agents. Ciprofloxacin, cefixime, polymyxin B, amoxicillin + sulbactam, and amoxicillin + clavulanic acid were the most sensitive drugs followed by amikacin, ofloxacin ampicillin, co-trimoxazole, and amoxicillin. Difference in antibiotic sensitivity was due to the resistance of microbe to different antibiotics and indiscriminate use of antibiotics [14]. Resistance may be due to spontaneously induced genetic mutation or the acquisition of resistance genes from other bacterial species by horizontal gene transfer via conjugation, transduction, or transformation. Many antibiotic resistance genes reside on transmissible plasmids, facilitating their transfer. Exposure to an antibiotic naturally selects for the survival of the organisms with the genes for resistance. In this way, a gene for antibiotic resistance may readily spread through an ecosystem of bacteria. Antibiotic-resistance plasmids frequently contain genes conferring resistance to several different antibiotics. Kumar *et al*. [14] also reported antibiotic resistant against different serotypes of *E. coli*. They concluded that *E. coli* strain isolated from diarrheic sheep/lambs belongs to a large number of serogroups and may be the reason for high variation in antibiotic resistance. However, present sensitivity pattern finding are not in confirmation with findings of Gaurav and Jakhar [13] and Kumar *et al*. [14] which could be ascribed to serotypes variation, acquired and genetic resistance, and selection of resistant microorganisms for different drugs.

## Conclusion

Major etiopathological cause of GIT disorders in sheep was *E. coli* infection, which causes a pathomorphological effect on various cadaver organs *viz*. abomasum, intestine, liver, mesenteric lymph nodes along with lesions on lungs, spleen, kidneys, and heart. *Haemonchus contortus* was the second most important causative agent for GIT disorders in sheep, which was responsible for weight loss, anemia, hemorrhagic enteritis, and hydropericardium. Ciprofloxacin, cefixime, polymyxin B, amoxicillin + sulbactam, and amoxicillin + clavulanic acid were the most sensitive drugs followed by amikacin, ofloxacin ampicillin, co-trimoxazole, and amoxicillin.

## Authors’ Contributions

SK and KKJ have designed the study and planned the research experiments. SK performed the research experiment and drafted the manuscript. KKJ revised the manuscript. VN and MP helped in conducting the experiment. All authors read and approved the final manuscript.
